# Impact of Initial Surgical Interventions on Empyema Outcomes: Insights From a Cohort Study in Yemen

**DOI:** 10.7759/cureus.69059

**Published:** 2024-09-10

**Authors:** Mohammed A Issa, Yasser A Obadiel, Khaled S Galeb, Haitham M Jowah

**Affiliations:** 1 Department of Surgery, Faculty of Medicine and Health Sciences, Sana'a University, Sana'a, YEM; 2 Department of Surgery, Republican Teaching Hospital Authority, Sana'a, YEM; 3 Department of Surgery, General Police Hospital, Sana'a, YEM

**Keywords:** complications, empyema, initial intervention, patient outcomes, pleural infection, thoracostomy drain tubes, thoracotomy

## Abstract

Purpose

The study aimed to evaluate the effectiveness of different initial interventions, including thoracostomy drain tubes, open thoracotomy with decortication, and video-assisted thoracoscopic surgery (VATS) thoracoscopy in the management of empyema.

Methods

This prospective cohort study was conducted at two teaching hospitals in Sana'a, Yemen, over a two-year period from 2022 to 2024. The study included 40 patients diagnosed with empyema, categorized according to the type of initial intervention received. Demographic data, clinical presentation, imaging findings, intervention details, and outcomes were systematically collected and analyzed. Statistical analyses were performed to identify associations between demographic characteristics, empyema stage, intervention type, and treatment success.

Results

The study included 40 patients with a higher proportion of males (67.5%) than females (32.5%). The mean age was 47.1 years (standard deviation (SD): 12.85). The overall success rate of the initial interventions was 55%, with significant variation based on empyema stage, comorbidities, and intervention type. Stage I empyema had the highest success rate (80%), followed by Stage II (50%) and Stage III (27.3%), with a statistically significant difference (p = 0.034). Smoking history was identified as a significant negative predictor of success (p = 0.001). Higher pleural fluid pH was associated with better outcomes (p = 0.015). The most common complications were chest infections (20%) and bronchopleural fistulas (10%), with a mortality rate of 7.5%.

Conclusion

The empyema stage significantly affects the success rate, with early stages showing better outcomes. Early and appropriate intervention, particularly in later stages, is crucial for better outcomes. Effective management of postoperative complications is vital. This study highlights the need for early diagnosis and tailored interventions based on the empyema stage to improve patient outcomes. Future research should focus on larger multicenter studies to validate these findings and develop standardized treatment protocols.

## Introduction

Empyema, defined as the accumulation of pus in the pleural cavity, is a significant clinical condition that often arises as a complication of pneumonia, thoracic surgery, trauma, or infection. The etiology of empyema typically involves bacterial infections, with *Streptococcus pneumoniae*, *Staphylococcus aureus*, and various anaerobic bacteria being the most commonly identified pathogens [1,2]. The clinical significance of empyema is underscored by its association with considerable morbidity and mortality, with studies reporting mortality rates of up to 20% among hospitalized patients [3]. Patients with empyema often present with fever, chest pain, and dyspnea, which can rapidly progress to severe sepsis and respiratory failure if not promptly addressed [4].

The management of empyema presents significant clinical challenges, primarily due to the complexity of its presentation and the necessity for timely and effective intervention. Early diagnosis and intervention are critical for improving patient outcomes, as delays can lead to increased morbidity and mortality [5]. Treatment modalities for empyema include thoracocentesis, chest tube drainage, video-assisted thoracoscopic surgery (VATS), and thoracotomy. Thoracocentesis involves the aspiration of pleural fluid using a needle, whereas chest tube drainage requires the insertion of a tube into the pleural space to continuously remove pus. VATS is a minimally invasive surgical technique that allows for the visualization and debridement of the pleural cavity, whereas thoracotomy is a more extensive surgical procedure involving an open chest approach [1,2].

Despite the variety of treatment options, considerable uncertainty remains regarding the optimal initial intervention for empyema. Studies have shown that the choice of first intervention can significantly influence patient outcomes, including the duration of hospital stay, complications, and overall survival rates [6]. For instance, a randomized controlled trial (RCT) by Rahman et al. (2011) demonstrated that intrapleural fibrinolytic therapy combined with chest tube drainage improved outcomes compared with chest tube drainage alone [6]. Similarly, Maskell et al. (2005) found that VATS was associated with better outcomes compared with chest tube drainage in patients with advanced empyema [7].

This prospective cohort study aimed to fill the knowledge gap by systematically evaluating the outcomes of various initial interventions for managing empyema. The primary objectives were to compare the efficacy and safety of thoracocentesis, chest tube drainage, VATS, and thoracotomy as first-line treatments for empyema, and to identify factors that may predict improved patient outcomes. By elucidating the impact of the initial intervention on the progression of empyema, this study sought to provide evidence-based recommendations for clinical practice, ultimately improving the standard of care for patients with this challenging condition.

This article was previously posted to the Research Square preprint server on August 13, 2024.

## Materials and methods

Study design

This prospective cohort study was designed to evaluate the outcomes of initial interventions for empyema. The cohort consisted of patients diagnosed with empyema who were treated at the Tertiary Republican Teaching Hospital Authority and General Police Hospital in Sana'a from 2022 to 2024. The study was approved by the Institutional Review Board (IRB) of the Faculty of Medicine and Health Sciences, Sana’a University (approval no.: IRB-FMHS/2021/0156), on November 10, 2021. Informed consent was obtained from all participants.

Study population

Participants were included in the study if they satisfied the following criteria: adults aged 18 years or older, diagnosed with empyema based on clinical presentation, imaging findings (chest X-ray and/or CT scan), and pleural fluid analysis (positive Gram stain or culture, or pus in the pleural space). They must have undergone one of the following initial interventions: thoracocentesis, chest tube drainage, VATS, or thoracotomy. Exclusion criteria included patients with a history of previous empyema, those who received more than one type of initial intervention simultaneously, and those with concomitant severe comorbid conditions that could independently influence outcomes (e.g., advanced malignancy, severe immunosuppression).

Data collection

Data were collected prospectively using standardized forms. The following information was recorded for each participant: demographic data (age, sex, comorbidities, and smoking status); clinical presentation (symptoms, duration of illness, and initial laboratory results including white blood cell count, glucose, and lactate dehydrogenase (LDH) levels); imaging findings (size and location of the empyema on chest X-ray and/or CT scan); and details of initial intervention (type of procedure - thoracostomy drain tubes, VATS, or thoracotomy with decortication - timing from diagnosis to intervention, and any complications during the procedure). Outcomes, such as duration of hospital stay, need for additional interventions, complications (e.g., pneumothorax, bleeding, infection), and mortality, were also recorded. Data collection was conducted by trained research personnel to ensure accuracy and consistency. All data were anonymized and securely stored to maintain patient confidentiality. Regular data audits were performed to ensure the completeness and accuracy of the collected information.

Interventions

Participants were categorized according to the type of initial intervention received: Thoracocentesis: pleural fluid is aspirated using a needle under ultrasound guidance; Chest tube drainage: the chest tube is inserted into the pleural space to continuously drain pus, guided by imaging techniques; VATS: minimally invasive surgical debridement of the pleural cavity using thoracoscopic equipment; Thoracotomy: an open surgical procedure involving an incision in the chest wall to access and debride the pleural cavity.

Outcome measures

The primary outcome measure was the success rate of the initial intervention. Success was defined as the clinical resolution of empyema and the absence of the need for additional surgical intervention. Secondary outcome measures included complication and mortality rates and the association of demographic characteristics and laboratory parameters with treatment success.

Statistical analysis

Data were analyzed using the Statistical Package for the Social Sciences (IBM SPSS Statistics for Windows, IBM Corp., Version 26.0, Armonk, NY). Descriptive statistics were used to summarize demographic and clinical characteristics, with continuous variables expressed as means ± standard deviations (SD) or medians with interquartile ranges, and categorical variables expressed as frequencies and percentages. Comparative analyses were conducted using the Chi-square test for categorical variables and the t-test for continuous variables. Multivariate logistic regression analysis was performed to identify factors independently associated with the success of the initial intervention. Kaplan-Meier survival curves were generated to compare mortality rates between different intervention groups, with log-rank tests used to assess statistical significance. A p-value of <0.05 was considered statistically significant for all analyses.

## Results

Demographic characteristics

The study included 40 patients diagnosed with empyema. The gender distribution revealed a higher percentage of males (67.5%) than females (32.5%). The age of the patients ranged from 19 to 75 years, with a mean age of 47.1 years and a median age of 50 years (SD: 12.85 years). The age distribution demonstrated that the majority of patients (58%) were middle-aged adults (36-55 years), followed by older adults (56-75 years), who represented 26% of the cohort, and young adults (19-35 years), who accounted for 16% (Table 1).

**Table 1:** Demographic Characteristics of the Study Participants

Characteristics	Frequency	Percentage
Gender		
Male	27	67.5
Female	13	32.5
Total	40	100.0
Age Group		
19-35	6	16
36-55	22	58
56-75	10	26
19-35	6	16
Age (Mean ± SD)	47.1±12.85	

Demographic characteristics and success rate

This study analyzed the association between demographic characteristics and the success rate of the initial intervention. Out of 40 patients, 22 were classified as successful and 18 as unsuccessful. The analysis revealed that age was not significantly associated with success rate (p-value = 0.248). In terms of gender, 77.3% of males (N=17) were successful compared with 55.6% (N=10) of unsuccessful males, while 22.7% of females (N=5) were successful compared with 44.4% (N=8) of unsuccessful females. Although there was a difference in success rates between genders, the difference was not statistically significant (p-value = 0.152). A notable finding was the strong association between smoking history and success rate. Among patients with a history of smoking (40.0%, N=16), only 18.2% (N=4) achieved success, whereas 66.7% (N=12) were unsuccessful. This association was statistically significant (p-value = 0.001). Regarding comorbidities, there were no significant associations with success rate for diabetes mellitus, hypertension, ischemic heart diseases, cardiovascular diseases, renal impairment, or other comorbidities (Table 2).

**Table 2:** Demographic Characteristics and Success Rate Among Study Participants Notes: The Chi-square test was used for categorical variables; * Statistical significance is indicated by p-value < 0.05. SD: standard deviation; DM: diabetes mellitus; HTN: hypertension; IHD: ischemic heart disease; CVD: cardiovascular disease

Category	Total (N=40)	Successful (N=22)	Unsuccessful (N=18)	P-value
Age (Mean±SD)	47.1/12.8	49.2/10.8	44.4/14.7	0.248
Gender				0.152
Male	27 (67.5%)	17 (77.3%)	10 (55.6%)	
Female	13 (32.5%)	5 (22.7%)	8 (44.4%)	
History of Smoking	16 (40.0%)	4 (18.2%)	12 (66.7%)	0.001*
Comorbidities				
DM	6 (15.0%)	3 (13.6%)	3 (16.7%)	0.796
HTN	13 (32.5%)	7 (31.8%)	6 (33.3%)	0.921
IHD	1 (2.5%)	1 (4.5%)	0 (0.0%)	0.373
CVD	2 (5.0%)	0 (0.0%)	2 (11.1%)	0.114
Renal Impairment	2 (5.0%)	0 (0.0%)	2 (11.1%)	0.114
Others	1 (2.5%)	1 (4.5%)	0 (0.0%)	0.373

Characteristics of the empyema and success rate

The study included 40 patients, of which 22 were classified as successful and 18 as unsuccessful. Regarding the side of the empyema, 80.0% (N=32) were on the right side, 15.0% (N=6) on the left side, and 5.0% (N=2) were bilateral. The success rates for each side were 81.8% (N=18) for the right side and 18.2% (N=4) for the left side. However, the association between the empyema side and success rate was not statistically significant (p-value = 0.136). In terms of the empyema stage, 12.5% (N=5) were classified as Stage I, 60.0% (N=24) as Stage II, and 27.5% (N=11) as Stage III. The success rates for each stage were 22.7% (N=5) for Stage I, 50.0% (N=11) for Stage II, and 27.3% (N=6) for Stage III. The association between the empyema stage and success rate was statistically significant, with Stage I cases showing a significantly lower success rate compared with the other stages (p-value = 0.034) (Table 3).

**Table 3:** Empyema Characteristics and Success Rate Among Study Participants Notes: The Chi-square test was used for categorical variables; * Statistical significance is indicated by a p-value < 0.05.

Category	Total (N=40)	Successful (N=22)	Unsuccessful (N=18)	P-value
Side				0.136
Right	32 (80.0%)	18 (81.8%)	14 (77.8%)	
Left	6 (15.0%)	4 (18.2%)	2 (11.1%)	
Bilateral	2 (5.0%)	0 (0.0%)	2 (11.1%)	
Stage				0.034*
Stage I	5 (12.5%)	1 (4.5%)	4 (22.2%)	
Stage II	24 (60.0%)	11 (50.0%)	13 (72.2%)	
Stage III	11 (27.5%)	10 (45.5%)	1 (5.6%)	

Laboratory characteristics

Several laboratory parameters of pleural fluid were measured. The mean pH values were 7.28 (SD=0.28) for all cases, 7.38 (SD=0.33) for successful cases, and 7.17 (SD=0.15) for unsuccessful cases. The correlation between pH and success rate was statistically significant (p-value = 0.015; t-test was used for continuous variables). Other laboratory measurements, including white blood cell count (WBC), glucose level, and LDH level, were not significantly associated with success rate. The presence of positive or negative cultures in the pleural fluid was not significantly associated with patient success rate (Table 4).

**Table 4:** Association Between Pleural Fluid Characteristics and Success Rate Notes: The Chi-square test was used for categorical variables, and ** indicates the t-test was used for continuous variables. Statistical significance is indicated by a p-value < 0.05. SD: standard deviation; WBC: white blood cell; LDH: lactate dehydrogenase

Category	Total (N=40)	Successful (N=22)	Unsuccessful (N=18)	P-value
pH (mean + SD)	7.28±0.28	7.38±0.33	7.17±0.15	0.015**
WBC (mean + SD)	3.48±1.60	3.48±1.69	3.47±1.54	0.992
Glucose (mean + SD)	46.45±5.86	47.32±6.16	45.39±5.46	0.306
LDH (mean +SD)	1410±667.8	1359±761.4	1472±547.5	0.601
Culture				0.283
Positive	10 (25.0%)	4 (18.2%)	6 (33.3%)	
Negative	30 (75.0%)	18 (81.8%)	12 (66.7%)	

Radiological findings

Radiological findings among the 40 cases included in the study revealed that the most prevalent finding was loculation/septation, which was observed in 29 cases (72.5% of patients). Encysted empyema was observed in nine cases (22.5%), pleural effusion in five cases (12.5%), pleural thickening in seven cases (17.5%), and cavitation in one case (2.5%) (Table 5).

**Table 5:** Radiological Findings Among Study Participants

Findings	Frequency	Percentage
Encysted Empyema	9	22.5
Pleural Effusion	5	12.5
Loculation/Septation	29	72.5
Pleural Thickening	7	17.5
Cavitation	1	2.5

Association between the first intervention and success rate

The study included 40 patients, of which 22 were classified as successful and 18 as unsuccessful. The first intervention options and their respective frequencies and percentages are as follows: VATS: three cases (7.5%); thoracostomy drainage tube: 23 cases (57.5%); thoracotomy + decortication: 14 patients (35.0%).

Among the successful cases, 9.0% (N=2) underwent VATS, 45.5% (N=10) underwent thoracostomy drainage tube, and 45.5% (N=10) underwent thoracotomy + decortication. Among the unsuccessful cases, 5.6% (N=1) underwent VATS, 72.2% (N=13) underwent thoracostomy drainage tube, and 22.2% (N=4) underwent thoracotomy + decortication. The association between the type of first intervention and success rate was not statistically significant (p = 0.307) (Table 6).

**Table 6:** Association Between First Intervention and Success Rate Notes: The Chi-square test was used for categorical variables. Statistical significance is indicated by a p-value < 0.05. VATS: video-assisted thoracoscopic surgery

Category	Total (N=40)	Successful (N=22)	Unsuccessful (N=18)	P-value
VATS	3 (7.5%)	2 (9.0%)	1 (5.6%)	0.307
Thoracostomy Drainage Tube	23 (57.5%)	10 (45.5%)	13 (72.2%)	-
Thoracotomy + Decortication	14 (35.0%)	10 (45.5%)	4 (22.2%)	-

Complications

The complications and mortality rates observed in the patients were documented. The most common postoperative complication was chest infection, accounting for 40% of the cases. Other complications included bronchopleural fistula (20%), surgical site infection (15%), and bleeding (10%). Mortality was observed in three cases (7.5%) (Table 7).

**Table 7:** Complications and Mortality Among Study Participants

Complications and Mortality Rates	Frequency	Percentage
Bronchopleural Fistula	4	10
Chest Infection	8	20
Thoracotomy Site Pain	6	15
Bleeding	3	7.5
Air Leak	3	7.5
Pneumothorax	1	2.5
Surgical Site Infection	5	12.5
Mortality	3	7.5

Correlations between stage, first intervention, outcome, and mortality

Table 8 presents the correlations between the stage of empyema, first intervention, outcome, and mortality. The study found that Stage I cases had a significantly lower success rate than other stages (p-value = 0.034; the result is statistically significant). The mortality rate was higher in patients who underwent thoracostomy drainage tube and thoracotomy + decortication compared to those who underwent VATS (Table 8).

**Table 8:** Correlations Between Stage, First Intervention, Outcome, and Mortality F: frequency or number of cases; VATS: video-assisted thoracoscopic surgery

Stage	F	First Intervention	F	Outcome			
				Success	Unsuccess	Mortality According to Stage	Mortality/First Intervention
Stage I	5	Thoracostomy Drainage Tube	4	4 (80%)	-	-	-
		VATS	1	1 (20%)	-	-	-
Stage II	24	Thoracostomy Drainage Tube	14	3 (12%)	9 (37.5%)	2 (8.3%)	14.3%
		Thoracotomy + Decortication	8	7 (29%)	1 (4%)	-	
		VATS	2	1 (50%)	1 (4%)	-	
Stage III	11	Thoracostomy Drainage Tube	5	1 (9%)	4 (36%)	-	16.7%
		Thoracotomy + Decortication	6	5 (45%)	-	1 (9.1%)	

## Discussion

The management of empyema remains controversial due to the significant influence of the initial intervention on patient outcomes, particularly when considering the empyema stage. This study evaluated the effectiveness of initial interventions, including thoracostomy drain tubes, open thoracotomy with decortication, and VATS thoracoscopy. Our results indicated that success rates varied depending on the empyema stage, comorbidities, and type of initial intervention, although complications were common and varied according to procedure and patient condition.

The study included 40 patients, with a higher proportion of males (67.5%) than females (32.5%). This gender distribution is consistent with several studies, such as Gupta et al. (2022) [8], which reported higher prevalence rates in males. Possible explanations for this disparity include higher rates of smoking and occupational exposure to respiratory irritants among males [9]. However, our results differ from Mitri et al. (2024) [10], who found a more balanced gender distribution.

Our study found no significant association between age and success rate (p = 0.248). Although males had a higher success rate, the difference was not statistically significant (p = 0.152). Smoking history was a significant negative predictor of success, which is consistent with the findings of Wozniak et al. (2009) [11] and Argento and Wahidi (2016) [12], who reported higher risks of complications and poorer recovery rates in smokers.

The empyema stage significantly impacted the success rate, with Stage I showing the highest success rate (p = 0.034). Early-stage empyema typically responds better to less-invasive treatment, leading to better outcomes. This finding supports the findings of Santana-Rodríguez et al. (2022) [13], who noted that early surgical intervention was correlated with better outcomes and reduced morbidity. Higher pH values were associated with better outcomes (p = 0.015), consistent with existing research. However, other laboratory parameters like WBC, glucose, and lactate levels did not show significant associations with success rates. This suggests that although these parameters are useful for diagnosing empyema, they may not strongly predict treatment outcomes on their own, as noted by Godfrey et al. (2019) [9].

The most common complications were chest infections (20%) and bronchopleural fistulas (10%). Surgical site infections (12.5%) and thoracotomy site pain (15%) were also significant concerns. Effective management strategies, including appropriate antibiotic use, careful surgical technique, and postoperative follow-up, are essential to minimize these risks. The mortality rate was 7.5%, which is within the expected range for empyema, considering the severity and complexity of the cases. Factors such as advanced age, comorbidities, and delayed intervention often contribute to mortality.

Among the successful cases, 9.0% underwent VATS, 45.5% underwent thoracostomy drainage tube, and 45.5% underwent thoracotomy + decortication. Most patients in Stage I received thoracostomy drainage tubes and VATS, with high success rates of 80% and 20%, respectively. These findings suggest that early-stage empyema can often be successfully managed with less invasive procedures. Minimally invasive techniques like VATS are effective in treating complicated parapneumonic effusions and empyema [14]. These results align with Mitri et al. (2024) [10], who found that early VATS is highly effective in managing pleural empyema, leading to quicker recovery, shorter hospital stays, and fewer complications.

Stage II patients had a varied distribution of first interventions. Thoracostomy drainage tube was the most commonly used drainage tube, but it had a lower success rate (12%) than thoracotomy and decortication (29.2%) and VATS (50%). This suggests that more invasive procedures are necessary for successful outcomes. Mortality in Stage II patients was 8.3%, with thoracotomy and decortication causing a mortality rate of 14.3%. This indicates that although thoracotomy and decortication are more effective in achieving success, they are associated with higher risks. In Stage III, thoracotomy and decortication had a higher success rate (45.4%) than thoracostomy drainage tube (9.1%). This finding underscores the need for more aggressive surgical interventions in the advanced stages of empyema. The mortality rate in Stage III was 9.1%, and thoracotomy and decortication were associated with a mortality rate of 16.7%. This finding reflects the increased complexity and risk of advanced empyema.

Our findings are consistent with those of previous studies that demonstrated the necessity of more invasive surgical interventions in the advanced stages of empyema. For example, a study by Karaman et al. (2004) [15] found that closed-tube thoracostomy was less effective in managing advanced empyema than open thoracotomy and decortication, particularly in more severe cases.

Similarly, Wozniak et al. (2009) [11] reported that thoracotomy and VATS were more successful initial procedures for managing Stage II empyema than tube thoracostomy, which often failed and required subsequent surgical intervention. This finding supports our observation that more invasive procedures, such as thoracotomy, decortication, and VATS, are necessary to achieve better outcomes in advanced stages.

A meta-analysis by Pan et al. (2017) [16] highlighted that while thoracotomy and decortication had higher success rates in advanced empyema, they were also associated with higher morbidity and mortality compared with VATS. This is consistent with our findings indicating higher mortality rates associated with thoracotomy and decortication, particularly in Stage III empyema.

Furthermore, a study by Chan et al. (2007) [17] comparing VATS and thoracotomy found that both approaches were equally effective in treating empyema, but VATS was associated with lower perioperative morbidity and mortality rates. This suggests that VATS is a preferable initial intervention for selected patients, particularly those with Stage II empyema, when clinically feasible.

Strengths and limitations of the study

The prospective design of this study offers significant advantages by reducing the number of biases commonly encountered in retrospective studies. Real-time data collection ensured the accuracy and consistency of patient information capture, which minimized recall bias and enhanced the reliability of our findings. This design allowed for comprehensive monitoring of patient responses and disease progression, thus strengthening the validity of the outcomes.

However, several limitations must be acknowledged. The lack of randomization introduces potential selection bias because treatment decisions were based on clinical judgment rather than randomized allocation. This may limit the generalizability of our findings because the patient population may not fully reflect a more randomized or diverse cohort. Furthermore, the small sample size reduced the statistical power of detecting subtle differences between interventions. Although significant associations were observed, a larger sample size would allow for a more nuanced analysis of the factors influencing treatment success.

Additionally, a single-center setting has the potential for bias because patient populations and clinical protocols at this center may not be representative of broader populations. Future research should consider conducting RCTs or multicenter studies with larger, more diverse cohorts to minimize bias and increase the generalizability of the findings.

## Conclusions

Our study showed that early-stage empyema responds better to less invasive procedures like VATS, although the small sample size and possible selection bias limit definitive conclusions. We found a significant association between smoking and poorer outcomes; however, confounding factors, such as advanced disease at presentation, may have influenced this result. Larger studies are needed to confirm these findings and explore the impact of smoking and other variables. Although our results are statistically significant, we urge caution in their clinical relevance, emphasizing the need for further research to refine empyema treatment strategies.
